# Charity—Limited

**Published:** 1892-03-05

**Authors:** 


### Passing Topics.

### CHARITY—LIMITED.

The very general application of the provisions of the Limited Liability Act to businesses until recently carried on as private concerns, under the personal supervision and direction of the principals, has had one unexpected and untoward result—the cessation of the firms' subscriptions to public charities. This was unexpected, because no reasonable man can suppose that serious thought would be taken by company promoters of the probable effect of any act of theirs upon benevolent organisations, and to that extent they are acquitted of a deliberate intention of injury; but the result is not the less untoward because in these hard times, rendered harder by the gradual extinction of the personal, and the exaltation of the official nd corporate element, it is becoming more difficult every year to replace lost helpers.

The Chairman of the Provident Clerks' Benevolent Fund, when he recently bewailed this fact, gave utterance to regrets which managers of other charitable institutions have already had occasion to feel when contemplating their subscription lists. In all likelihood there is not a hospital in London of any magnitude which could not furnish a formidable list of contributions withdrawn for no better reason in each case than that the concern has been turned into a limited company; and in order to guage the full measure of the loss, it must be remembered that the sources of income now run dry have been among the most prolific. City liberality has been proverbial, and we have known many an astute secretary who, whether the occasion were a festival, a meeting, or merely an every-day search for treasure, would infinitely prefer the co-operation of a plain City man before that of the whole peerage, whose august members, with some honoured and notable exceptions, are apt to consider the loan of a name a sufficient contribution to any undertaking whatever. Many of the great mercantile princes have been at once munificent contributors and active workers, bringing to the assistance of the institution not only goodly gifts, but a share of their business capacity and a co-operation which concerned itself in enlisting fresh recruits among their friends, a task rendered easier by the force of their own example.

And now, in place of ready sympathy and accessible hearts, we have to deal with a veritable *chevaux de frise*—the articles of association—behind which the board of directors sit obdurate. That, at least, is the picture suggested by the speaker we have referred to, and if it be a fact, that without a specific permission accorded by the articles directors of companies cannot perform their duty in regard to public charity, is it too much to ask them to take steps to emancipate themselves from a position we cannot believe the majority of shareholders would desire they should occupy, and which, if it does enable them to retain, what is at most an inappreciable portion of revenue, at the same time excludes them from the enjoyment of a time-honoured privilege.

It seems to us that the honour of our great commercial and financial houses is involved, and that it is altogether unworthy of themselves and of their traditions that the announcement they "have no funds from which to contribute," already far too frequent, should become stereotyped. A phrase of this kind in the mouths of those who represent bodies distributing tens or hundreds of thousands of pounds annually, has not the sterling City ring about it we were at one time accustomed to. Agricola.

